# Treadmill exercise rescues motor deficits in parkinsonian mice by modulating striatal D2-MSN activity: evidence from calcium imaging and chemogenetics

**DOI:** 10.3389/fnsys.2026.1851618

**Published:** 2026-06-12

**Authors:** Ping Chen, Yuan Zeng, Bing Liu, Quan Yang, Wan Chun Xue, Nanlin Liu, Chunyu Zhuang

**Affiliations:** 1 College of Physical Education and Science, Jishou University, Jishou, China; 2 Department of Nursing, Haikou Maternal and Child Health Hospital, Haikou, China

**Keywords:** behavioral dysfunction, dopamine type-2 receptor, exercise, medium spiny neurons, Parkinson’s disease, striatum

## Abstract

**Objective:**

The aim of this study is to investigate whether treadmill exercise alleviates motor dysfunction in a mouse model of Parkinson’s disease (PD) by modulating the excitability of striatal medium spiny neurons expressing dopamine type 2 receptors (D2-MSNs).

**Methods:**

A unilateral 6-hydroxydopamine (6-OHDA) injection was performed in the right striatum of D2-Cre mice to establish a hemi-lesioned PD model, with sham-operated mice serving as controls. PD mice were subjected to treadmill exercise (18 m/min, 40 min/day, 5 days/week for 4 weeks). Motor function was evaluated using the open-field test, rotarod, and negative geotaxis test. The excitability of D2-MSNs was assessed via fiber-photometric calcium imaging (ΔF/F, AUC, and peak amplitude), Western blotting for c-Fos protein expression, and double immunofluorescence labeling of D2R and c-Fos. Furthermore, chemogenetic approaches (hM4Di-Gi for inhibition and hM3Dq-Gq for activation) were employed to validate the causal role of D2-MSN excitability in exercise-mediated motor recovery.

**Results:**

PD mice exhibited significant motor deficits, characterized by reduced locomotor activity, shortened latency to fall on the rotarod, and increased turning latency in the negative geotaxis test. Calcium imaging and c-Fos expression analyses revealed a marked hyperexcitability of striatal D2-MSNs in PD mice compared to controls (*p* < 0.01). Treadmill exercise significantly attenuated this D2-MSN hyperexcitability and concurrently improved all motor performance metrics (*p* < 0.01). Chemogenetic inhibition of D2-MSNs mimicked the beneficial effects of exercise in PD mice, whereas chemogenetic activation of these neurons abolished the exercise-induced motor improvements and reversed the reduction in neuronal excitability (*p* < 0.01).

**Conclusion:**

Our findings demonstrate that striatal D2-MSN hyperexcitability is a critical pathological feature of motor dysfunction in PD mice. Treadmill exercise rescues motor deficits by suppressing this hyperexcitability. These results provide novel insights into the neurobiological mechanisms underlying the therapeutic benefits of physical exercise in Parkinson’s disease.

## Introduction

Parkinson’s disease (PD) is a typical neurodegenerative disorder characterized mainly by motor dysfunction. Patients not only experience typical motor symptoms such as tremors, bradykinesia and rigidity, but also are accompanied by non-motor symptoms such as sleep disorders and cognitive impairments. The characteristics of this disease, such as its long course and difficulty of treatment, greatly affect patients’ quality of life (Spix et al., 2021). A report published in The Lancet Neurology also shows that PD is the fastest-growing neurodegenerative disease in terms of prevalence, disability rate and mortality rate (GBD 2016 Parkinson’s Disease Collaborators, 2018). Up to now, neither drugs nor surgery can only improve the symptoms of patients, but cannot stop the progression of the disease, let alone cure PD. Therefore, exploring new therapeutic strategies and cellular and molecular targets remains the core task of current PD research.

The basal ganglia (BG) are the primary subcortical region of the central brain, playing a pivotal role in motor regulation. Structurally and functionally, it can be divided into five nuclei: the striatum, the lateral part of the globus pallidus, the compact part of the substantia nigra, the subthalamic nucleus, and the complex of the reticular part of the substantia nigra/medial part of the globus pallidus. The cortico-basal ganglia-thalamo-cortical neural circuit is involved in the planning, initiation, and execution of movements, as well as the acquisition of new motor skills (Wang M. et al., 2022; Wang X. et al., 2022). As the core of BG, the striatum integrates excitatory inputs from the cortex and thalamus, coordinates voluntary movements, as well as motor learning and decision-making. These functions mainly rely on medium spiny neurons (MSNs), which account for about 95% of striatal neurons. Based on the connections between brain regions and chemical phenotypes, striatal MSNs can be divided into two subgroups: namely, MSNs on the direct pathway (dMSNs) and MSNs on the indirect pathway (iMSNs). The axons emitted by the dMSNs project directly to the output nucleus of the BG, namely the globus pallidus internus/substantia nigra pars reticulata complex (GPi/SNr). It mainly expresses dopamine I receptors (D1R) coupled with Gαs/Gαolf proteins, substance P and endorphins (hence dMSNs are also called D1-MSNs). In contrast, iMSNs first projects to the outer part of the globus pallidus (GPe), mainly expressing dopamine II receptors conjugated with Gαi/Gαo proteins. D2R and enkephalin (hence iMSNs is also called D2-MSNs) (Suarez et al., 2020). The classical theory holds that activating D1-MSNs facilitates movement, while activating D2-MSNs inhibits the generation of redundant movements. The two pathways jointly regulate precise movement (Gerfen, 2022). In the PD state, the DAergic neurons in the substantia nigra pars compacta (SNpc) undergo progressive degeneration, leading to a decrease in the DA concentration of the striatum. This results in a reduction in the activity of D1-MSNs and a significant increase in the activity of D2-MSNs (i.e., an imbalance in the functions of the direct and indirect pathways). The net effect of this phenomenon is to increase the electrical activity of the SNr/GPi, thereby inhibiting the target nuclei that receive projection from the basal ganglia, which are located at the base of the brain. According to Zhang et al. (2021), the primary cause of PD-related behavioral dysfunction is the inhibition of movement.

Epidemiological investigation results show that individuals who frequently engage in physical activities in early adulthood are associated with a lower risk of developing PD in their later years (Crotty and Schwarzschild, 2020). Clinical research has confirmed that physical therapy using different movement patterns is both effective and feasible in the rehabilitation of PD patients, and can significantly improve behavioral functional disorders such as gait, balance, and coordination in PD patients (Feng et al., 2020). As demonstrated in previous laboratory studies, a 4-week moderate-intensity treadmill training intervention has been shown to selectively reduce the loss of D2-MSN dendritic spines in the striatum of PD model rats (Ping et al., 2020). This intervention has also been shown to significantly alleviate behavioral dysfunction, including autonomous activity ability and motor coordination, in PD model rats. Literature reports that after treatment with an appropriate amount of Levodopa (L-DOPA) or D2R agonists, the excitability of D2-MSNs decreases, and the behavioral and functional abnormalities of PD patients are alleviated (Servillo et al., 2024; Wei et al., 2013). Based on our previous research and the existing literature, we hypothesized that exercise may alleviate PD-related behavioral dysfunction by reducing the excitability of D2-MSNs in the striatum. To test this hypothesis, the present study established a PD model using D2-Cre transgenic mice. By comprehensively applying fiber optic calcium imaging, chemogenetics, molecular biology, and behavioral approaches, we sought to verify that exercise alleviates PD-related behavioral dysfunction by reducing the excitability of striatal D2-MSNs. It provides a new perspective for the study of the neurobiological mechanism by which exercise improves PD-related behavioral dysfunction.

## Materials and methods

### Main experimental reagents

rAAV-EF1α-DIO-GCaMP6s-WPRE-hGHpA, rAAV-EF1α-DIO-hM4D(Gi)-mCherry- WPRE, rAAV-EF1α-DIO-hM3D(Gq)-mCherry-WPRE, rAAV-EF1α-DIO-mCherry- WPRE-hGHpA and Clozapine N-oxide (CNO) [PT0071, PT0043, PT0042, PT0013 and CNO-01, BrainVTA (Wuhan)]. 6-Hydroxydopamine (6-OHDA) (H4381, Sigma); Apomorphine (APO) (PHR2621, Sigma); Tyrosine hydroxylase (TH) (AB152, Sigma); Dopamine type 2 receptor (D2R) (BS75097, Bioworld); Cellular proto-oncogene Fos (c-Fos) (Ab208942, Abcam).

### Experimental animals and grouping

SPF-grade D2-Cre transgenic male mice [C57BL/6J-Tg(Drd2-Cre), Stock No. GAP1059] (generated using BAC transgenic technology, in which a Cre cassette was inserted upstream of the ATG start codon of the BAC Drd2 gene, allowing Cre recombinase expression to be driven by the endogenous promoter and regulatory elements of the Drd2 gene present on the BAC clone, thereby specifically targeting dopamine D2 receptor-expressing neurons in the striatum, on a C57BL/6J genetic background) (aged 8–10 weeks, weighing 22–25 g) were purchased from Amygdala (Guangzhou) Biotechnology Co., Ltd. (Production License No.: SCXK (E) 2021–2025).

Mice were raised under a 12-h light–dark cycle, provided with freely accessible water and food. Both the animal care and experimental plans have been approved by the school’s Laboratory Animal Ethics Committee (No: JSDX-2022-0008). After 1 week of environmental adaptation and treadmill adaptation training, they were randomly divided into the sham operation Control group and the 6-OHDA model group. Mice that were identified as meeting the PD model were randomly divided into the PD quiet group (PD), the PD + exercise group (PD + Ex), the PD + chemogenetic inhibition group (PD + hM4Di), and the control group (PD + mCherry) and PD + exercise+chemogenetic excitation group (PD + Ex + hM3Dq) and its control group (PD + mCherry).

### Mouse genotype identification

DNA extraction was carried out on mouse tail samples using the mouse tail genomic DNA extraction kit. Genotypes were identified by PCR detection.

1) Using the obtained whole-genome DNA of mice as a template, primers were designed based on the transgenic types of mice, and the corresponding PCR reaction system was configured.
2) The prepared PCR reaction solution was added to the PCR tube, which was then placed in the PCR instrument and run according to the preset amplification program to achieve DNA amplification.
3) A 1% agarose gel was prepared. A volume of 10 μL of the PCR amplification product was loaded into the sample wells of the gel, and electrophoresis was performed for 30 min at a constant voltage of 110 mV.
4) The gel was subsequently imaged using a gel electrophoresis analysis system. The genotype of each mouse (positive, negative, or heterozygous) was determined by assessing the presence or absence of the target band on the gel.

### Stereotactic surgery

The mice were anesthetized with isoflurane (3–4% for induction, maintained at 1–2% in oxygen) via inhalation and subsequently fixed on a stereotactic apparatus. Referring to the modeling method of Wang M. et al. (2022) and Wang X. et al. (2022), 6-OHDA (2 μg/μL, dissolved in 0.9% sodium chloride aqueous solution containing 0.2% ascorbic acid) was stereotactically injected into the right striatum at two sites (4 μg per site, 8 μg total) using a LEGATO130 micro-injection pump (RWD Life Science Co., Ltd., China) connected to a microsyringe. The stereotactic coordinates relative to bregma were as follows: AP + 0.5 mm, L + 2.0 mm, DV -3.0 mm (site 1) and DV -2.0 mm (site 2) (Paxinos and Franklin, 2012). A total volume of 2 μL was injected at each site at a rate of 0.5 μL/min. The needle should be retained for 5 min after each injection. The sham operation group was injected with the same dose of normal saline containing 0.2% ascorbic acid at the same site.

For fiber photometry recording, an adeno-associated virus (AAV) vector, rAAV-EF1α-DIO-GCaMp6s-WPRE-hGHpA, encoding the calcium concentration -sensitive protein GCaMP6s, was stereotactically injected into the right striatum at a volume of 300 nL and a rate of 20 nL/min. The stereotactic coordinates relative to bregma were as follows: AP + 1.0 mm, ML + 2.4 mm, DV -2.65 mm (Paxinos and Franklin, 2012). The needle should be retained for 10 min after each injection. Following the completion of the virus injection process, the subsequent step involves the insertion of the optical fibre ceramic ferrule into the same position as the virus injection.

Chemogenetic viruses were stereotaxically injected into the right striatum (AP: +0.5 mm, ML: +1.8 mm, DV: −3.0 mm relative to bregma). Specifically, one of the following viral vectors was injected per subject: rAAV-EF1α-DIO-hM4D(Gi) -mCherry-WPRE, rAAV-EF1α-DIO-hM3D(Gq)-mCherry-WPRE, or rAAV-EF1α -DIO-mCherry-WPRE-hGH polyA (serving as the control vector). Each virus was infused at a volume of 300 nL at a rate of 20 nL/min. The needle should be retained for 10 min after each injection.

The surgical wound was sutured and 200,000 U/d of penicillin was injected continuously for 3 days after the operation to prevent infection.

### Identification of PD models

Seven days after 6-OHDA injection, the APO-induced rotation experiment was conducted on mice. Apomorphine (APO) solution (0.125 g/L, 0.35 mL/kg) was administered subcutaneously, and the number of complete rotations was recorded for 30 min beginning 5 min post-injection. The difference in the number of rotations of the mouse to the healthy side (left) and the injured side (right) exceeding 120 turns was taken as the basis for determining the successful preparation of the PD mouse model (Wang et al., 2025). Three mice from the 6-OHDA model group and three from the sham operation group were randomly selected for striatal TH immunohistochemical staining to further verify the reliability of the model.

### Exercise intervention program

One week after the operation, the mice in the exercise group were subjected to treadmill exercise for 4 consecutive weeks. The training protocol was: 18 m/min, 40 min/day, 5 days/week (rest on Saturdays and Sundays) (Zhao et al., 2020). The mice in the non-exercise group were placed on a treadmill for the same period of time but did not perform treadmill exercises.

### Optical fiber photometric recording

Mice were placed in a dark environment, and the fluorescence signals of the target brain regions were monitored in real time using a fiber optic fluorescence photometric system equipped with a 473 nm excitation light, a 505–544 nm emitter filter, and a highly sensitive photomultiplier tube detector. The analog voltage signal was digitized at 100 Hz and recorded using the Power1401 digital converter and Spike 2 software. When setting up the connection link between the optical fiber implantation and the photometric detection system, a dedicated optical fiber equipped with an integrated rotary joint should be selected. This type of optical fiber can automatically adapt to the torsional and tensile forces generated by the movement of experimental animals by taking advantage of the flexible steering performance of the rotary joint, thereby effectively avoiding damage such as fiber breakage and wear caused by animal movement. Not only that, this dedicated optical fiber, with its excellent compatibility, ensures a stable connection between the optical fiber and the photometric system while minimizing the impact on the normal activities of experimental animals to the greatest extent. During the experiment, to reduce the photobleaching effect, the laser power at the end of the optical fiber needs to be precisely regulated and set within a low power range of 20–40 μW. The collected calcium signals are processed by digital filtering through analysis software and then quantitatively analyzed using the △F/*F* value.

### Behavioral tests

On the day of the behavioral test, the mice were transferred to the test room and had to adapt to the indoor conditions (odor, temperature, light and background noise, etc.) for ≥30 min before the experiment began. Mice were treated with normal saline or CNO (IP in 2 mg/kg body weight normal saline) 30 min before the test (Saline or CNO was administered only once on the day of behavioral testing, following the completion of the 4-week exercise intervention.). The experiment was conducted in a dark and quiet environment to minimize external interference.

### Open field experiment

The open field test was conducted in an open box (40 × 40 × 40 cm), the floor of which was divided into a central zone (20 × 20 cm) and a peripheral zone. After the mice adapted to the laboratory environment, they were gently placed in the center of the open field box, and the free activities of the mice for 5 min were recorded using the ANY-Maze automatic video tracking software. After the behavioral tests were completed, the behavioral video data of the experimental animals were quantitatively analyzed using the SMART 3.0 behavioral analysis system that came with the experimental equipment. Immediately after each mouse experiment is completed, the open field experimental site should be thoroughly cleaned with 75% alcohol. Once the ethanol had fully evaporated and the apparatus was dry, the subsequent mouse was introduced for testing (Li et al., 2025).

### Rotating rod experiment

Motor coordination and balance were assessed using a 5-channel rotarod apparatus (1.25-inch diameter). Prior to behavioral testing, all experimental animals underwent adaptive training over three consecutive days, with two sessions per day separated by a 2-h inter-session interval. On the first training day, mice were placed on a stationary rod for 2 min, followed by rotation at a constant speed of 5 rpm for 2 min. On the second training day, the rod was maintained at a constant speed of 11 rpm for 3 min per session. On the third training day, the rod was maintained at a constant speed of 22 rpm for 3 min per session. For PD model mice that fell from the rod within 1 min, a single re-placement onto the rod was permitted. On the test day, mice were first subjected to a constant speed of 5 rpm for 5 min, followed by a linear acceleration phase in which the rod accelerated from 5 rpm to 40 rpm over 300 s. The latency to fall (s) was recorded as the primary outcome measure. Between each trial, the rotarod apparatus was thoroughly cleaned with 75% ethanol and allowed to dry completely before the subsequent animal was tested (Laverne et al., 2022; Nielsen and Ford, 2024).

### Negative geotactic experiment

The experimental apparatus consisted of an inclined platform fixed at a 45° angle, constructed from a checkered acrylic sheet mounted on a base acrylic sheet. Each mouse was placed at the top of the platform in a head-down position and was held in place for 5 s before being released. The time required for the mouse to complete a 180° turn (from head-down to head-up) was recorded and denoted as the ‘turning latency.’ Each mouse was tested three times, with an inter-trial interval of no less than 5 min. If a mouse touched the side wall or fell off the platform, the trial was repeated (with a maximum of two additional attempts permitted). The mean of the three valid turning latencies was taken as the final result for each animal. Between each trial, the platform was wiped with 75% ethanol and allowed to dry completely before the subsequent animal was tested (Penny et al., 2021).

### Western blotting (WB) experiment

The expression level of c-Fos protein in the striatum was detected by Western blotting. Total protein was extracted using RIPA lysis buffer, and protein concentration was quantified using a BCA kit. A total of 20 μg of protein per sample was separated by 10% SDS-PAGE and wet-transferred onto a polyvinylidene fluoride (PVDF) membrane at 100 V for 90 min. The membrane was blocked in 5% skimmed milk-TBST at room temperature for 1 h, followed by incubation with mouse anti-c-Fos antibody (1:1000, ab208942) overnight at 4 °C on a rocking shaker. The following day, the membrane was washed three times with TBST (5 min per wash), then incubated with HRP-conjugated goat anti-mouse IgG secondary antibody (1:10,000, BE0102) at room temperature for 1 h, followed by three additional TBST washes (5 min per wash). Protein bands were visualized using an ECL chemiluminescence detection system, and images were acquired using a chemiluminescence imaging system. Band intensity was analyzed using ImageJ, and the relative expression level of each target protein was normalized to the internal reference protein GAPDH, expressed as the ratio of integrated optical density (IOD).

## Detection of TH and c-Fos expression levels

### Detection of TH expression levels in the striatum

The density of TH-immunopositive fibers in the striatum was assessed by immunohistochemical staining. Mice were deeply anesthetized via isoflurane inhalation (3–4%), and adequate depth of anesthesia was confirmed by the complete absence of the pedal withdrawal reflex and loss of response to noxious stimuli. Animals were then immediately transcardially perfused with 20 mL of 0.9% normal saline, followed by slow perfusion fixation with 50 mL of 4% paraformaldehyde solution. The brain was carefully dissected out and post-fixed in 4% paraformaldehyde for 24 h at 4 °C. The brain tissues were subsequently dehydrated through a graded ethanol series (70, 80, 90, 95, and 100%), cleared in xylene, and embedded in paraffin wax. The striatum was localized by reference to the mouse brain stereotaxic atlas, and tissue blocks were trimmed accordingly prior to embedding. Continuous coronal sections were cut at a thickness of 5 μm, and every third section was selected for staining. Sections were baked at 60 °C for 2 h, then deparaffinized in xylene and rehydrated through a graded ethanol series. Antigen retrieval was performed by immersing sections in 0.01 M sodium citrate buffer (pH 6.0) using a water bath. Endogenous peroxidase activity was quenched by incubation in 3% H₂O₂-PBS at room temperature for 15 min, followed by three washes in PBS (3 min each). Sections were then blocked at room temperature for 30 min, followed by overnight incubation at 4 °C in a humidified chamber with rabbit anti-TH primary antibody (1:3000). The following day, sections were washed three times with PBS (3 min per wash) and incubated with HRP-conjugated goat anti-rabbit IgG secondary antibody (1:500) at room temperature for 1 h, followed by three additional PBS washes (3 min per wash). Immunoreactivity was visualized using a DAB chromogenic solution, and sections were subsequently coverslipped. Images were acquired using an Axio Scope 5 microscope and analyzed with the associated built-in software.

### Detection of c-Fos expression level on striatal D2-MSNs

The co-expression of c-Fos and D2 receptors in striatal D2-MSNs was assessed by immunofluorescence double-labeling staining. Mice were deeply anesthetized via isoflurane inhalation (3–4%), and adequate depth of anesthesia was confirmed by the complete absence of the pedal withdrawal reflex and loss of response to noxious stimuli. Animals were then immediately transcardially perfused with 20 mL of 0.9% normal saline, followed by slow perfusion fixation with 50 mL of 4% paraformaldehyde solution. The brain was carefully dissected out and post-fixed in 4% paraformaldehyde for 24 h at 4 °C. Brain tissues were subsequently dehydrated through a graded ethanol series (70, 80, 90, 95, and 100%), cleared in xylene, and embedded in paraffin wax. The striatum was localized by reference to the mouse brain stereotaxic atlas, and tissue blocks were trimmed accordingly prior to embedding. Continuous coronal sections were cut at a thickness of 5 μm, and every third section was selected for staining. Sections were baked at 60 °C for 2 h, then deparaffinized in xylene and rehydrated through a graded ethanol series. Antigen retrieval was performed by immersing sections in 0.01 M sodium citrate buffer (pH 6.0) using a water bath. Sections were then blocked with 5% normal serum in PBS at room temperature for 30 min to minimize non-specific binding, followed by overnight incubation at 4 °C in a humidified chamber with a mixture of mouse anti-c-Fos (1:1000) and rabbit anti-D2R (1:1000) primary antibodies. The following day, sections were washed three times with PBS (3 min per wash) and incubated with a mixture of Alexa Fluor 594-conjugated goat anti-rabbit IgG (1:500, red) and Alexa Fluor 488-conjugated goat anti-mouse IgG (1:500, green) secondary antibodies at room temperature for 1 h, protected from light. Sections were subsequently washed three times with PBS (3 min per wash), followed by incubation with DAPI (1 μg/mL) at room temperature for 5 min in the dark. After three additional PBS washes (3 min per wash), sections were coverslipped using an anti-fluorescence quenching mounting medium and stored at 4 °C protected from light. Images were acquired using a Zeiss Axio Scope 5 fluorescence microscope and analyzed with the associated built-in software.

### Data statistical processing

The data were statistically analyzed using SPSS 26.0 statistical software package and GraphPad Prism 6 software was used for plotting. For the mean values of the indicators, the K-S test is used to test whether they conform to the normal distribution, and the F-test is used to test the homogeneity of variance at the same time. Measurement data conforming to the normal distribution were expressed as Mean± standard deviation (Mean ± SD). Multiple comparisons among multiple groups were conducted using one-way analysis of variance (ANOVA), and Tukey *post hoc* tests were selected for inter-group comparisons. The difference in the number of rotations between the sham operation group and the model group was analyzed using the independent sample *t*-test. A *p* value <0.05 was regarded as statistically significant.

## Results

### Evaluation of PD mouse models

**Figure 1:**
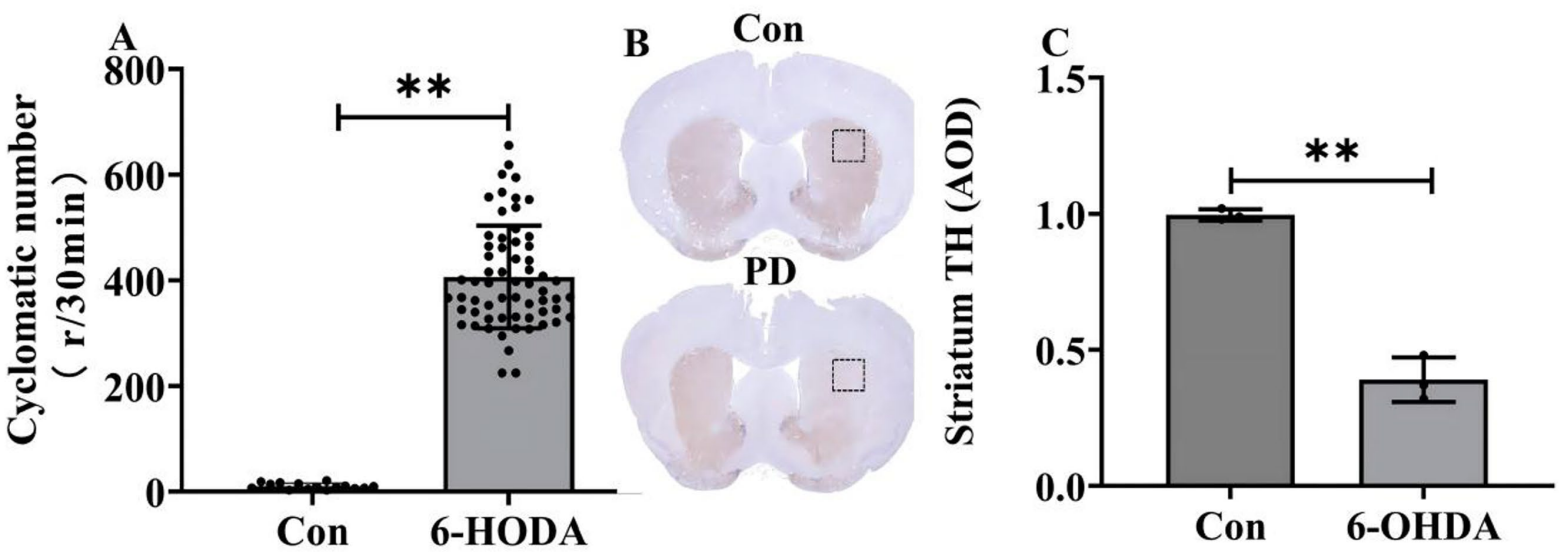
Results of APO-induced rotation behavior test and striatal TH immunohistochemical staining. **(A)** Comparison of apomorphine (APO)-induced rotations in mice (Control, *n* = 15; 6-OHDA, *n* = 59). **(B)** Representative images of tyrosine hydroxylase (TH) immunohistochemical staining in the striatum (*n* = 3). **(C)** Comparison of the mean optical density of striatal TH-immunopositive fiber terminals (*n* = 3). AOD, Average optical density. **p < 0.01.

The results of the APO-induced rotation behavior test showed that all 6-OHDA -lesioned mice met the model criteria (rotation count > 100 turns/30 min) (Figure 1A). To further validate the reliability of the model, three mice from the 6-OHDA group meeting the inclusion criteria (>100 rotations/30 min) and three mice from the Sham group were randomly selected for striatal tyrosine hydroxylase (TH) immunohistochemical staining. The results demonstrated that, compared with the Control group, the mean optical density of TH-immunopositive fiber terminals in the lesioned striatum of the 6-OHDA group was significantly reduced (*p* = 0.001) (Figures 1B,C).

### Exercise reduces the excitability of striatal D2-MSNs in PD mouse models

**Figure 2:**
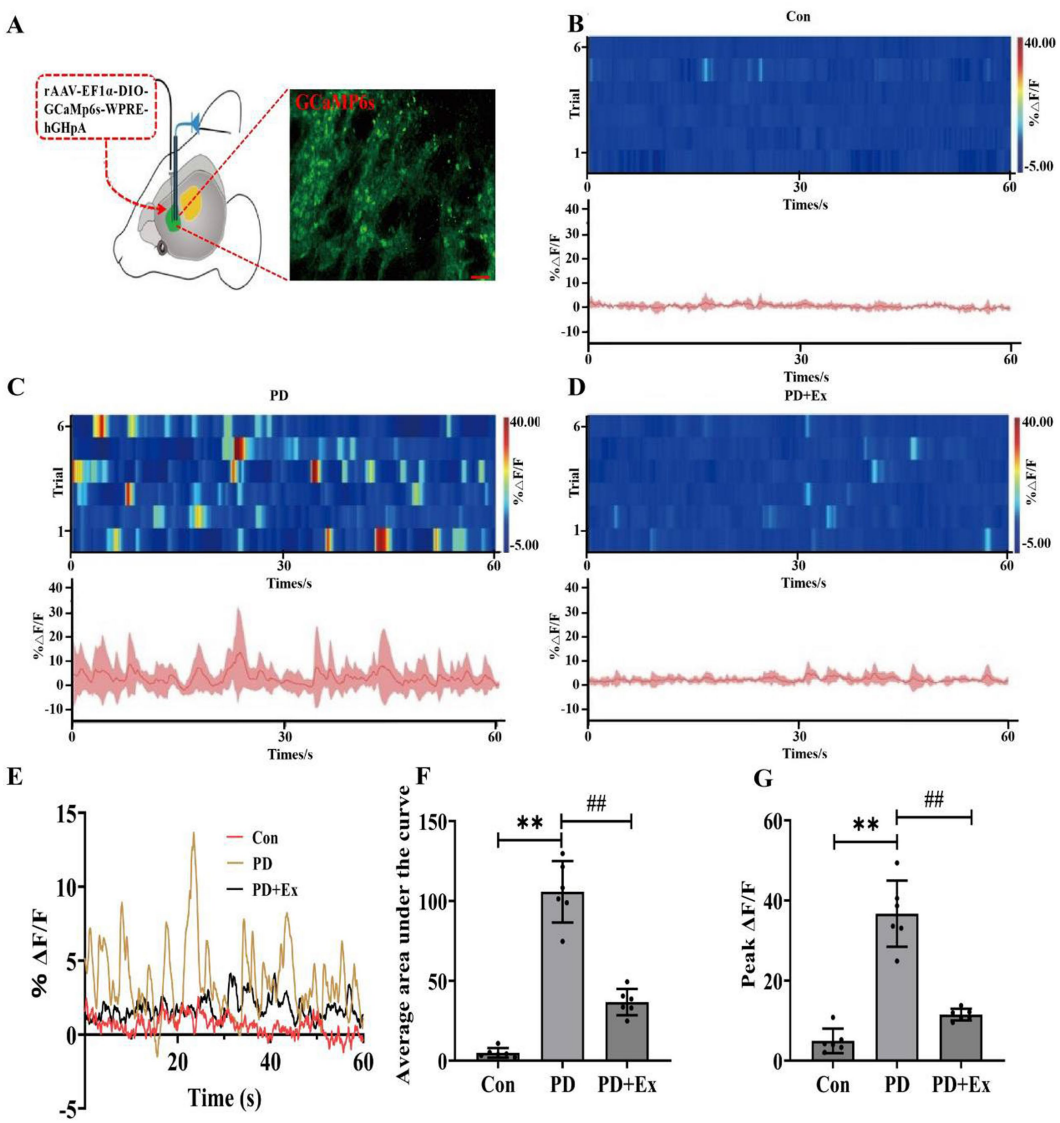
Results of excitability detection of striatal D2-MSNs in mice from different groups. **(A)** Left panel: Schematic showing the injection of rAAV-EF1α-DIO-GCaMP6s into the striatum of D2-Cre mice. Right panel: Representative images of GCaMP6s expression in the striatum of D2-Cre mice (20×). Scale bar: 50 μm. **(B–D)** Calcium transient heatmaps and ΔF/*F* value plots of striatal D2-MSNs in mice from different groups (*n* = 6); **(E)** Comparison of ΔF/F of calcium transients in striatal D2-MSNs among different groups (*n* = 6); **(F)** Comparison of AUC of calcium transients ΔF/F in striatal D2-MSNs among different groups (*n* = 6); **(G)** Comparison of peak values of calcium transients ΔF/F in striatal D2-MSNs among different groups (*n* = 6). Compared with the control group, **p < 0.01; compared with the PD group, ##*p* < 0.01.

Fiber photometry recording results showed that, compared with the Control group, the area under the curve (AUC) and peak (Peak) of calcium transients ΔF/F in striatal D2-MSNs of PD mice were significantly increased [(AUC): *p* = 0.001; (Peak): *p* = 0.000]. Compared with the PD group, the AUC and Peak of calcium transients ΔF/F in striatal D2-MSNs of PD + Ex mice were significantly decreased [(AUC) and (Peak): *p* = 0.000] (Figures 2A–G).

### Exercise alleviates behavioral functional impairments in PD mouse models by reducing the excitability of striatal D2-MSNs

**Figure 3:**
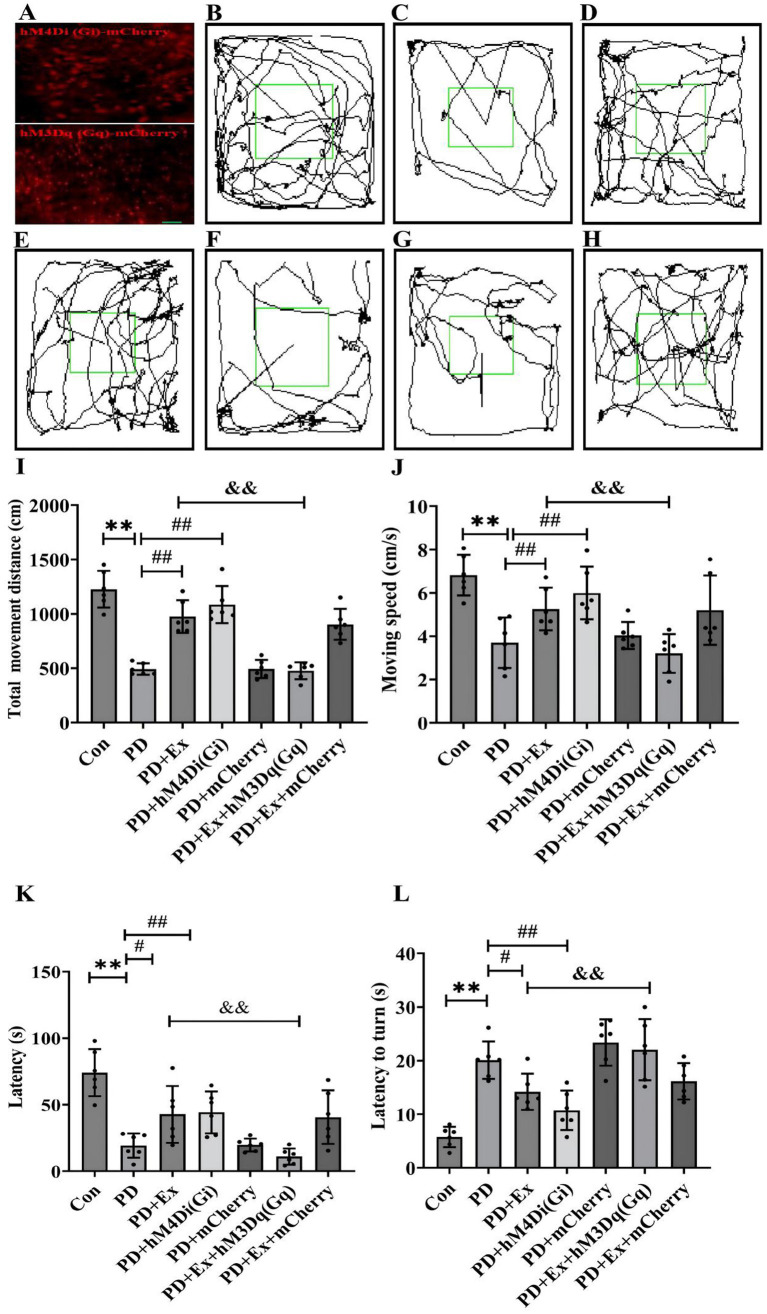
Comparison of spontaneous activity and motor coordination in mice from different groups. **(A)** Representative images showing the expression of chemogenetic viruses in striatal D2-MSNs (20×). Scale bar: 50 μm. **(B–H)** Locomotor trajectories of mice from control, PD, PD + Ex, PD + hM4Di(Gi), PD + mCherry, PD + Ex + hM3Dq(Gq), and PD + Ex+ mCherry groups in the open field box (*n* = 6). **(I)** Comparison of total distance traveled in the open field among different groups (*n* = 6). **(J)** Comparison of average locomotor speed in the open field among different groups (*n* = 6). **(K)** Comparison of time spent on the rotarod among different groups (*n* = 6). **(L)** Comparison of turning delay time in the negative geotaxis test among different groups (*n* = 6). Compared with the control group, **p < 0.01; compared with the PD group, #*p* < 0.05, ##*p* < 0.01; compared with the PD + Ex group, &&*p* < 0.01.

Open field test results showed that, compared with the Control group, PD mice exhibited a significant decrease in total distance traveled and average speed in the open field (*p* = 0.000). Compared with PD mice, both PD + Ex and PD + hM4Di(Gi) (PD + chemogenetic inhibition) mice showed a significant increase in total distance traveled (*p* = 0.000) and average speed (*p* = 0.009; *p* = 0.001). Compared with PD + Ex mice, PD + Ex + hM3Dq(Gq) (PD + Ex+chemogenetic excitation) mice had a significant decrease in total distance traveled and average speed (*p* = 0.000; *p* = 0.001) (Figures 3A–J).

Rotarod test results showed that, Compared with the Control group, PD mice had a significant reduction in the time spent on the rotarod (*p* = 0.000). Compared with PD mice, both PD + Ex and PD + hM4Di(Gi) mice showed a significant increase in the time spent on the rotarod (*p* = 0.01; *p* = 0.006). Compared with PD + Ex mice, PD + Ex + hM3Dq(Gq) mice had a significant decrease in the time spent on the rotarod (*p* = 0.001) (Figure 3K).

Negative geotaxis test results showed that, compared with the Control group, PD mice exhibited a significant increase in turning delay time (*p* = 0.000). Compared with PD mice, both PD + Ex and PD + hM4Di(Gi) mice showed a significant decrease in turning delay time (*p* = 0.012; *p* = 0.006). Compared with PD + Ex mice, PD + Ex + hM3Dq(Gq) mice had a significant increase in turning delay time (*p* = 0.001) (Figure 3L).

### Exercise downregulates c-Fos protein expression in striatal D2-MSNs of PD mouse models

**Figure 4:**
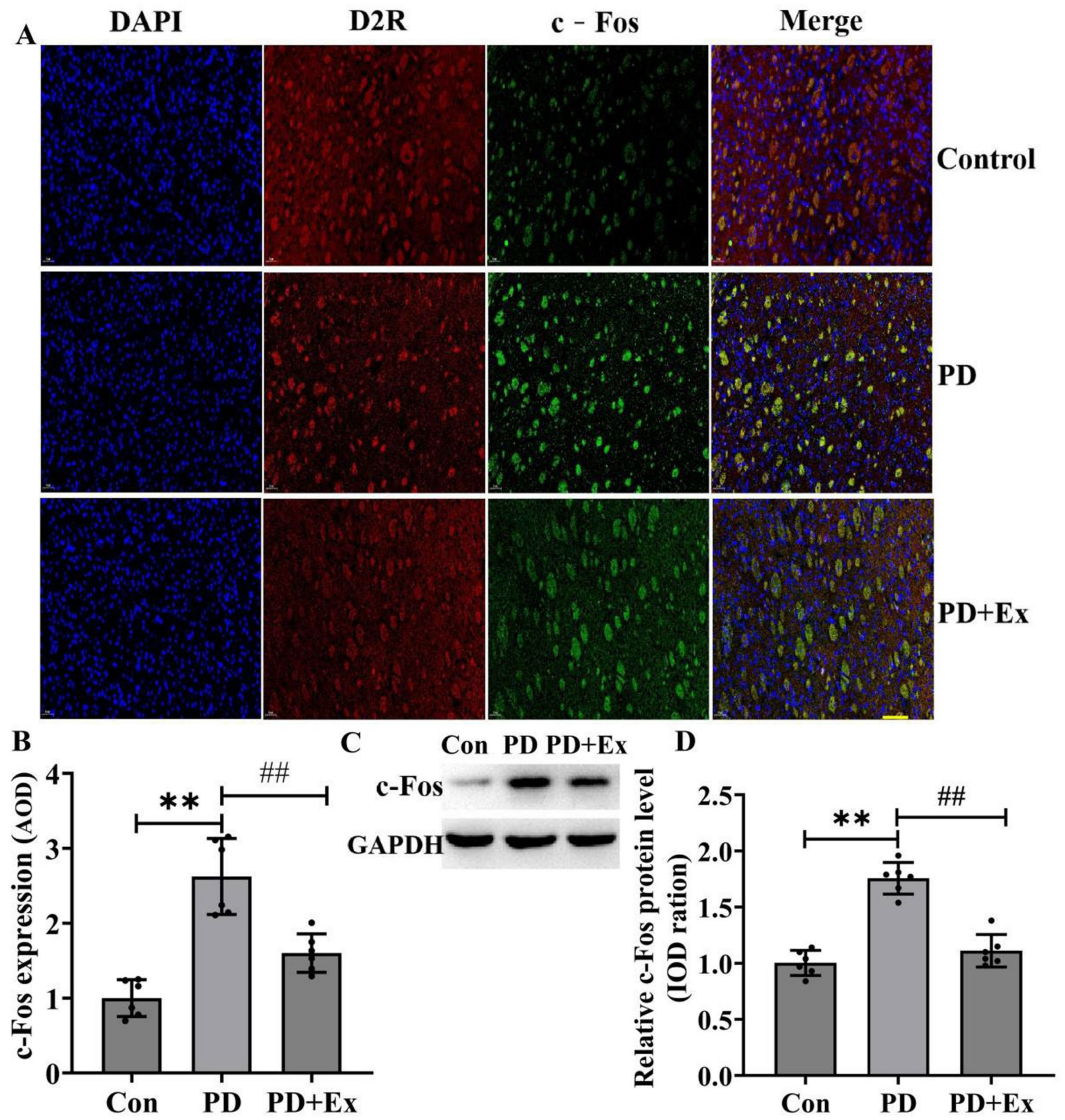
Comparison of c-Fos protein expression levels in the striatum of mice from different groups. **(A)** Immunofluorescence double labeling staining results of D2R and c-Fos in the striatum of mice from different groups (20×) (*n* = 6). Scale bar: 50 μm. **(B)** Comparison of average optical density of c-Fos among different groups (*n* = 6). **(C)** Western blot results of c-Fos protein expression in the striatum of mice from different groups (*n* = 6). **(D)** Comparison of c-Fos protein expression levels among different groups (*n* = 6). IOD: Integrated optical density. Compared with the control group, **p < 0.01; compared with the PD group, ##*p* < 0.01.

Immunofluorescence double labeling staining results showed that, Compared with the Control group, the expression level of c-Fos protein in striatal D2-MSNs of PD mice was significantly upregulated (*p* = 0.000). Compared with PD mice, the expression level of c-Fos protein in striatal D2-MSNs of PD + Ex mice was significantly downregulated (*p* = 0.000) (Figures 4A,B).

Western blot (WB) detection results showed that, compared with the Control group, the expression level of c-Fos protein in the striatum of PD mice was significantly upregulated (*p* = 0.000). Compared with PD mice, the expression level of c-Fos protein in the striatum of PD + Ex mice was significantly downregulated (*p* = 0.000) (Figures 4C,D).

## Discussion

This study for the first time confirmed in D2-Cre transgenic PD mice that 4-week moderate-intensity treadmill training significantly improved motor dysfunction in PD model mice by reducing the excitability of D2-MSNs in the striatum. Fiber optic calcium imaging records showed that exercise intervention significantly reduced the transient AUC and Peak of calcium D2-MSNs in PD model mice. The chemogenetic inhibition/activation technique has been demonstrated to achieve “on–off” control over the exercise benefits, thereby providing causal evidence that “D2-MSNs are key cellular nodes of exercise effects”.

### PD striatum D2-MSNs hyperexcitation

The “direct pathway/indirect pathway” model proposed in the late 1980s has greatly promoted the understanding of the basal ganglia (BG) circuit, PD pathophysiology, and treatment strategies for BG-related movement disorders (McGregor and Nelson, 2019). Studies in animal models of PD have demonstrated that striatal dopamine (DA) depletion leads to an imbalance in the activity of the two pathways—specifically, an increase in GABAergic striatal output from indirect pathway neurons and a decrease in striatal output from direct pathway neurons—thereby enhancing basal ganglia inhibition of the thalamo-cortical motor system and giving rise to the characteristic motor symptoms of Parkinson’s disease (Albin et al., 1989; Delong, 1990; Obeso et al., 2000). Parker et al. (2018) employed an Inscopix miniscope for freely moving calcium imaging to monitor the activity of striatal MSNs in a PD mouse model. They found that striatal DA depletion resulted in an imbalance between D1-MSN and D2-MSN activity, manifesting as a significant decrease in D1-MSN activity rate and a significant increase in D2-MSN activity rate compared to pre-lesion levels—both at rest and during locomotion—throughout the progression from acute (day 1 post-6-OHDA lesion) to long-term (day 14 post-6-OHDA lesion) DA depletion. Wang et al. (2024) confirmed by using optogenetics combined with *in vivo* multi-channel electrophysiological techniques that the discharge frequency of D2-MSNs in the dorsal striatum of PD model mice was significantly higher than that of the control group mice. Zhao et al. (2022) selected D2-Cre mice as the research subjects. After conducting whole-cell patch clamp recording of D2-MSNs in the stripe, they found that the frequency and amplitude of spontaneous excitatory postsynaptic current (sEPSC) in D2-MSNs of PD model mice were significantly increased. Belluscio et al. (2019) found that even after partial degeneration of the substantia nigra striatum pathway, the excitability of D2-MSNs would increase.

Fiber optic photometry is an optical technique that takes advantage of the strict correspondence between changes in calcium ion concentration and neuronal activity. By using special fluorescent dyes, the concentration of calcium ions in neurons is expressed through fluorescence intensity and captured by the fiber optic recording system, thereby achieving the purpose of detecting neuronal activity. In recent years, fiber optic photometry has been widely applied in the field of neurology research to observe the activities of specific types of neurons (Simpson et al., 2024). In this study, the excitability of D2-MSs was quantified by optical fiber photometric recording of the calcium sensor GCaMP6s encoded by the gene expressed in the striatum of D2-Cre mice. The results showed that compared with the Control group, the area under the ΔF/F curve (AUC) and Peak value (Peak) of calcium transient ΔF/F in the striatum D2-MSNs of mice in the PD group were significantly increased, indicating that the striatum D2-MSNs of PD model mice were overexcited. c-Fos is a protein encoded by the immediate early gene. The upregulation of its expression is usually associated with enhanced neuronal activity and is used as a molecular marker for neuronal activation (Witzig et al., 2023). Therefore, in this study, the detection by immunofluorescence dual-labeling staining method also showed that the expression level of c-Fos protein on D2-MSNs in the striatum of mice in the PD group was significantly upregulated compared with that in the Control group, and was consistent with the WB examination results of c-Fos protein in the striatum. It was further confirmed that the excitability of D2-MSNs in the striatum of PD model mice was enhanced. Combining previous studies, whether it is *in vivo* or ex vivo electrophysiological detection, or direct/indirect calcium imaging or molecular biological detection, it has been confirmed that the degeneration of substantia nigro-striatum DAergic neurons in the PD state can increase the excitability of striatum D2-MSNs (Wang et al., 2024; Zhao et al., 2022; Simpson et al., 2024).

### “Modulating” the excessive excitation of D2-MSNs in the striatum using motor stimuli alleviates behavioral dysfunctions in mouse models of PD

As an auxiliary and alternative non-pharmaceutical treatment strategy, the positive role of various types of exercise therapy (such as aerobic exercise, gait training, balance training, progressive resistance training and psychosomatic exercise) in improving PD-related behavioral dysfunction (including balance, gait, fall risk and physical function) has received increasing attention (Ernst et al., 2023). Previous laboratory studies have shown that a 4-week moderate-intensity treadmill training intervention can significantly reduce the number of rotations in PD model rats, and significantly increase the total distance, total exercise time and average exercise speed in the open field (Chen and Li, 2019). However, the exact neurobiological mechanism through which exercise improves PD-related behavioral dysfunction has not been fully clarified yet. Literature reports that after treatment with an appropriate amount of L-DOPA or D2R agonists, the excitability of D2-MSNs decreases, and the behavioral and functional abnormalities of PD patients are alleviated (Wei et al., 2013). Further evidence indicates that D2R agonists can significantly improve motor dysfunction, gait abnormalities, and postural imbalance in MPTP- or 6-OHDA-induced PD mouse models, reduce the excitability of striatal D2-MSNs, and suppress cAMP production in D2-MSNs (Li et al., 2023). Liu et al. (2020) and Zhao et al. (2022) employed optogenetic techniques to express photosensitive proteins in the striatal D2-MSNs of D2-Cre transgenic mice. By precisely applying light stimulation of the corresponding wavelength, they achieved spatiotemporally precise modulation of the neuronal activity of D2-MSNs. The results revealed that optogenetic inhibition of striatal D2-MSNs can significantly increase the total movement distance and average movement speed of PD model mice in the open field. Grimm et al. (2021) demonstrated that optogenetic activation of unilateral striatal D2-MSNs in normal mice could significantly increase the number of rotations and significantly increase the angle at which the head turned to one side. Because the response wavelengths of photosensitive proteins are mostly short-wave visible light with poor penetration, optical fibers need to be implanted for deep brain region stimulation, which can cause brain damage and inflammatory responses (Li et al., 2025). Chemogenetics, which specifically activates or inhibits the activity of neurons in deep brain regions without the need for optical fiber implantation in a non-invasive manner, has been widely applied in research areas such as signal transduction, drug development, and functional genomics. Therefore, in this study, chemogenetic techniques were employed to precisely regulate the dorsal striatum D2-MSNs of D2-Cre mice, and its impact on PD-related behavioral dysfunction was observed. The results show that 4 weeks of moderate-intensity treadmill training can significantly improve the ability of PD model mice to perform autonomous activities and coordinate their movements and balance. Specifically, the total moving distance and moving speed in the open field are significantly increased, the latency of falling from the spinning bar is significantly prolonged, and the turning delay time in the negative approach to the ground experiment is significantly shortened. Chemogenetic inhibition of striatal D2-MSNs in PD model mice can significantly increase the total moving distance and speed of the mice in the open field, significantly increase the time they stay on the rotating bar, and significantly reduce the turning delay time. However, chemogenetic excitation of D2-MSNs in the striatum of PD + Ex mice caused the positive effects of movement to disappear, manifested as a significant reduction in the total moving distance and moving speed in the open field, a significant decrease in the time spent on the rotating bar, and a significant increase in the turning delay time. Combining the effects of previous studies on the regulation of striatal D2-MSNs on the improvement of PD-related behavioral dysfunction, it is further confirmed that exercise improves the behavioral dysfunction of PD model mice by “correcting” the excessive excitation of striatal D2-MSNs.

## Conclusion

The excitability of D2-MSNs in the striatum of PD model mice was significantly enhanced; Exercise can significantly reduce the excitability of D2-MSNs in the striatum of PD model mice, and significantly improve motor coordination and autonomous activity ability. However, chemogenetic manipulation of D2-MSNs can eliminate the reducing effect of exercise on the excitability of D2-MSNs in the striatum of PD model mice and the alleviating effect on behavioral dysfunction. It indicates that the beneficial effect of exercise in improving behavioral dysfunction in PD model mice is achieved by reducing the excitability of striatal D2-MSNs. This study not only provides strong evidence for exercise as a non-pharmaceutical treatment strategy, but also offers new directions for future clinical applications. Future research should further explore the long-term effects of exercise on the excitability of D2-MSNs, as well as the signaling pathways (such as the RGS9-2/cAMP/NMDAR axis) and molecular mechanisms involved in the impact of exercise on the excitability of D2-MSNs.

## Data Availability

The raw data supporting the conclusions of this article will be made available by the authors, without undue reservation.
